# The Effect of Lubricant on the Detorque Force of Locator/Kerator Abutment in Overdenture Patients: A Pilot Clinical Study 

**DOI:** 10.30476/dentjods.2024.101754.2316

**Published:** 2025-06-01

**Authors:** Ali Chamani, Davood Aghasizadeh, Hamidreza Rajati Haghi, Ghazal Aghebati, Reza Shakiba

**Affiliations:** 1 Postgraduate Student, Dept. of Endodontics, Faculty of Dentistry, Mashhad University of Medical Sciences, Mashhad, Iran.; 2 Dept. of Prosthodontics, Faculty of Dentistry, Mashhad University of Medical Sciences, Mashhad, Iran.; 3 Student Research Committee, Faculty of Dentistry, Mashhad University of Medical Sciences, Mashhad, Iran.

**Keywords:** Dental Implantation, Dental Abutments, Lubricants, Torque

## Abstract

**Background:**

Despite the implants' remarkable success, mechanical failure of implant-abutment screws is a challenge for clinicians. Among the types of mechanical failures, abutment screw loosening is still frequently reported in the literature.

**Purpose:**

The present study aimed to evaluate the effect of using lubricant on the detorque force (removal force) of locator/kerator abutment in overdenture patients after 6 months of clinical usage.

**Materials and Method:**

This pilot clinical study examined the detorque force of implants in 20 edentulous patients with two mandibular implants positioned between the mental foramen. The patients underwent the fabrication of a complete maxillary prosthesis and mandibular overdenture. Before loading attachments in the delivery session, healing abutments were removed, and implant interiors dried, randomly some fixtures received a tetracycline eye ointment 1% lubricant, while the others remained non-lubricated. Abutments were secured with prescribed torque according to implant system guidelines (20 N.cm). After 6 months, detorque forces for the abutment locators were measured using a torque meter. Descriptive statistics, mean comparison, and Pearson correlation were performed on the obtained data. A significant level of *p* Value<0.05 was considered in the present study.

**Results:**

The mean detorque force was 13.4±1.94 in the non-lubricated group and 16.35± 1.24 in the lubricated group, which was significantly higher in the lubricated group (*p*< 0.001). Other results of the study include the non-significance of the difference in detorque force within male and female groups (*p*> 0.05), as well as the non-significance of Pearson's correlation between patients' age and detorque force (*p*> 0.05).

**Conclusion:**

According to the results, the use of lubricant significantly increases the detorque force, and it is recommended to use tetracycline eye ointment as a lubricant in implant treatment processes.

## Introduction

Using implants to restore complete or partial edentulism has improved chewing function, increased patient satisfaction compared to removable or fixed dental prostheses, and ultimately increased the patients' life quality [ 1 - 2 ]. These developments have made the implant a treatment choice in the patients' treatment plan [ 3 ]. Despite the implants' remarkable success, mechanical failure of implant-abutment screws is a challenge for clinicians. Among the types of mechanical failures, abutment screw loosening is still frequently reported in the literature [ 4 - 5 ]. This issue is clinically very critical because many cases it is not possible to remove the crowns intact, and the preload stress leads to mechanical failure during screw opening. On the other hand, loosening of the crown may lead to oblique forces entering the implant-abutment site and screw failure . In general, it is estimated that the incidence of abutment screw loosening includes 6.7% of cases [ 8 ]. However, this prevalence is different according to the type of implant-based prosthesis [ 6 ]. 

The forces that enter the screw joint can be divided into two general categories. First, the forces that keep the parts involved in the screw joint together (clamping forces).Second, the forces that, when applied to the screw joint, tend to separate the components and move them away from contact with each other (separating forces) [ 9 ]. Screw loosening will occur if either the separation forces are more than the clamping forces or when the pre-load is lost [ 10 ]. 

It is reported that lubrication increases the preload of the screw and therefore will reduce the screw loosening in the future [ 11 ]. However, this issue has not been investigated in overdenture patients in the literature. This subject is important in these patients because the full function of patients will be more dependent on implants than a single-unit implant or fixed partial dentures. [ 12 ]. For this reason, this study was conducted to investigate the effect of lubricant on the fixture's inner surface on the amount of force required to open locator/kerator abutments in overdenture patients. 

## Materials and Method

### Study design 

This study is a three-way blinded clinical trial (patients, operator, and analyzer). The screws of the implants were randomly divided into two lubricated and non-lubricated groups for patients. In addition, in the present study, it has been tried to comply with CONSORT 2010 guidelines [ 13 ].

### Sample size calculation

Because this study is being conducted on humans for the first time, it was directed as a pilot study based on the available resources and patients, and eventually 20 volunteers participated in the study.

### Ethical considerations and participants

This study was conducted on 20 completely edentulous patients who had two implants placed in the lower jaw in the area between the mental foramen and were ready to load. Informed consent was obtained from the patients to participate in the study. In this study, all Declarations of Helsinki were followed. This study was approved by the Ethics Committee of Mashhad University of Medical Sciences with the code of IR.MUMS. DENTISTRY.REC.1398.082. 

Patients over 18 years of age with complete edentulous jaws who were candidates for overdenture type 1 (OD-1) treatment were included in the study between 2019 and 2022. Patients with uncontrolled systemic disease, patients with neurological disorders and head and neck cancers, patients with tooth-supported overdenture, angled implants, or implant asymmetries from the midline, and patients with peri-implant bone loss were excluded from the study.

### Treatment protocol

The fabrication of a complete upper jaw prosthesis and overdenture prosthesis supported on tissue and mandibular implants went to the final stage, and ultimately, the attachments were installed chairside in the delivery session. Before installing attachments, healing abutments were opened, and with isolation conditions, the inner surface of the implants was completely air-dried, and one of the fixture surfaces was lubricated with a 1% tetracycline eye ointment (Sina Darou, IRAN) using a syringe. The fixtures were numbered and then randomly selected by the randomizer software to use the lubricant. Based on this, the patients were divided into two groups: Lubricant and Non-lubricant groups.

### Data acquisition

The locator abutments, which were selected with the appropriate gingival height (GH), were closed and torqued with an implant torque wrench (BioHorizons, USA) with a force of 20 N.cm. This wrench breaks after applying a force equal to 20 N/cm and minimizes the force error applied. After installing the attachments and the necessary adjustments, the prostheses were delivered to the patients, and the required follow-up sessions were conducted. Then, after six months of clinical use of dentures, the patients were called, and the required force to detorque (open) both locators of the abutments was measured and recorded using the torque meter (M-ark 10 corporation-USA). During force measurement, it was tried to minimize possible measurement errors by controlling the weight of the device and high accuracy.

**Figure 1:**
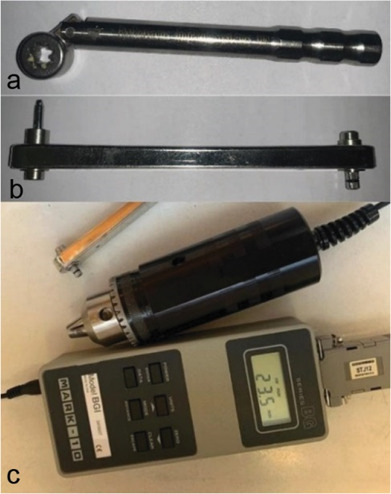
The instruments used in order; **a:** Implant torque wrench, **b:** Implant torque wrench to torque meter converter, and **c:** Torque meter

To easily measure the detorque force in the patient's mouth, a converter was used, one end was connected to the implant wrench inside the patient's mouth, and the other was connected to the torque meter outside the patient's mouth. In Figure 1, a view of the devices used in the present study is illustrated in order. The operator was blind to the fixture conditions, and the data was analyzed by a third person who was blind to the groupings (Figure 1). 

### Statistical analysis

The data obtained from the patients were processed using SPSS software version 25 (IBM, SPSS Inc, USA). Descriptive and frequency statistics were reported for quantitative and qualitative data. After checking the normality of the data, comparative statistics were measured by paired t-test and independent t-test. Pearson's correlation coefficient was also used to measure the relationship of the resulting data. In the present study, assuming a type I error of 5%, a significance level of *p*< 0.05 was considered. 

## Results

A total of 20 patients were enrolled at the beginning of the study, and two patients dropped out during the follow-up stages, eventually, this study was conducted with 18 patients, including seven women (38.9%) and 11 men (61.1%), with a mean age of 57.3±11.1 years and an age range of 49 to 64 years.

Due to the normal data distribution (*p*> 0.05), parametric statistics were used. The mean detorque force in the state without lubricant was 14.01±1.98 in women and 13.15±1.92 in men, which had no significant difference (*p*= 0.370). Also, the mean detorque force in the state with lubricant was 16.36±1.28 in women and 16.35±1.27 in men, and still no significant difference was observed (*p*= 0.98) (Table 1). 

**Table 1:** Comparison of mean detorque forces in both cases with and without lubricant by gender

Variable		Result	*p* Value^*^
Detorque force without lubricant	Male	13.15±1.92	0.370^1^
	Female	14.01±1.98	
Detorque force with lubricant	Male	16.35±1.27	0.985^1^
	Female	16.36±1.28	

* The significance level of P&lt;0.05 was considered

1. Independent t-test

However, the comparison of the mean detorque force in the two groups with lubricant and without lubricant showed a significant difference based on gender separation (*p*< 0.001). Apart from gender separation, in total (men+women), it was also shown that the detorque force with lubricant is significantly higher than the detorque force without lubricant (*p*< 0.001) (Table 2).

**Table 2:** Comparison of mean detorque forces between cases with and without lubricants by gender and in total

Groups		Result	*p* Value^*^
Male	Detorque force without lubricant	13.15±1.92	<0.001^1^
	Detorque force with lubricant	16.35±1.27	
Female	Detorque force without lubricant	14.01±1.98	0.001^1^
	Detorque force with lubricant	16.36±1.28	
Total	Detorque force without lubricant	13.48±.94	<0.001^1^
	Detorque force with lubricant	16.35±1.24	

* The significance level of p< 0.05 was considered

1. Paired t-test

**Figure 2:**
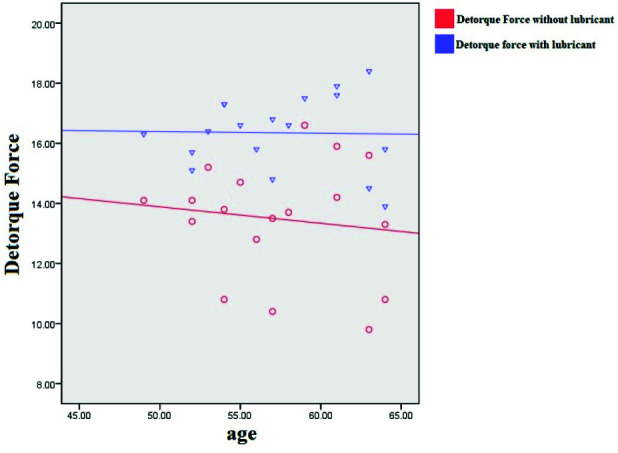
Distribution and linear correlation between age and detorque force separately with and without lubricant

According to Pearson's correlation coefficient test, the correlation of detorque forces with age in both cases with and without lubricant showed that it has a weak and inverse relationship, but none of the correlation values were significant (without lubricant: r= -0.13, *p* =0.608/ with lubricant: r= -0.021, *p*= 0.935) (Figure 2). 

## Discussion

Loose and unstable prosthetic screws may result in grave issues, including the breakage of the screw, the loosening of the prosthesis, or implant failure [ 14 ]. 

Preload is a contributing factor in the stability of the-screw and implant components, which depends on factors such as torque application device, type of screw and abutment alloy, implant type design, implant surface, and lubrication [ 15 ].

According to the present study, the mean detorque force in the group without lubricant was 13.48±1.94, and in the group with lubricant was 16.35±1.24, which statistically significantly increased in the group with lubricant (p&lt; 0.001). Schulte and Coffey [ 16 ] acknowledged that the reverse torque is 80% of the initial torque. In the present study, the mean detorque force in the lubricant group was approximately 80% of the initial torque, and the mean detorque in the non-lubricant group was approximately 67% of the initial torque force. It should be noted that in the mentioned study [ 16 ], no load was placed on the fixtures, on the other hand, in the present study, the detorque force was measured after 6 months of clinical use, based on the load resulting from chewing.

By reducing friction with lubricant, more force can be applied to induce preload. In the study of Vallee *et al*. [ 17 ], it was reported that 90% of the torque entered during the tightening phase of a screw joint is used for the first time to overcome friction, and only 10% of it is used to induce preload. As in the present study, these results were obtained.

Nigro *et al*. [ 18 ] also compared and investigated the detorque force in lubricated (smeared by artificial saliva) and non-lubricated groups. It was found that 15% of torque was lost in the non-lubricated group, and only 1% of torque force was lost in the lubricated group, so the detorque force was significantly higher in the lubricated group. The alignment of the present study and the mentioned study [ 18 ] shows that lubrication is directly related to initial torque force and causes higher preload. This phenomenon prevents the screw loosening. Duarte *et al*. [ 19 ] also showed that the detorque force in abutment screws increases after exposure to artificial saliva during 90 days, which is consistent with the results of the present study.

When the abutment is loaded, micro-movements occur at the abutment-implant junction, causing the surface irregularities and micro-roughness of the screw surface and connecting surfaces to be smoothed. In this way, the more surface irregularities and the less compatibility of components after casting, the more wear occurs between the contact surfaces after periodic loading. This wear brings the metal surfaces closer together so that this phenomenon (screw-retained) is reported to reduce the initial preload by 10-20% [ 20 - 21 ]. In the present study, it was observed that the detorque force was higher in cases where lubricant was used. Therefore, the screw head was more compatible with the implant and abutment.

However, studies whose results are inconsistent with the present study have also been reported in the literature [ 11 , 22 ]. Norton MR *et al*. [ 23 ] reported that the amount of detorque force in titanium screw abutments was not increased by impregnation in saliva. In their study [ 23 ], artificial saliva mixed with Xylitol 1.5% and heated to 37°C was used as a lubricant *in vitro* on the inner surface of the fixtures. The results showed no significant difference in detorque force in the non-lubricated and lubricated states. The reason for the inconsistent results of this study with our study can be attributed to the difference in the torque meter accuracy, and the different nature of the *in vitro* and clinical studies. In addition, the use of artificial saliva, whose quality and ability to make lubricants is uncertain, can be one of the reasons for the difference in the results with the present study. 

As mentioned above, the type of lubricant also affects the preload force. In studies, different results were reported regarding the effect of artificial saliva on detorque force [ 18 - 19 , 23 ]. Mariana de Almeida *et al*. [ 24 ]; investigated the effect of Vaseline on the screw-joints stability in implant abutments. The results showed that the use of Vaseline on the inner surface of the fixtures as a lubricant does not have a significant effect on maintaining the preload force and preventing screw loosening. The Vaseline viscosity is higher than the lubricant used in the present study (1% tetracycline eye ointment). It can be possible that not all the threads on the entire inner surface of the fixture have been coated with it. In the mentioned study [ 24 ], it was stated that Vaseline did not significantly reduce the friction between the screw and the fixture to increase the preload. Moreover, to increase preload and detorque force, in addition to the type of lubricant used, the material type of screw used is also important. It has been shown in previous studies that threads coated with gold increase the preload force [ 14 , 25 ]. In general, the literature reviews have shown that screws with modified alloys that have a lower friction coefficient; can lead to an increase in the preload force between the threads [ 26 ].

In a systemic review study by Nithyapriya *et al*. [ 27 ], it has been demonstrated that the use of dry lubricants containing 60-80 nm nanoparticles as well as human saliva helps to maintain the preload force and increasing the screw-fixture stability by reducing the friction coefficient. In addition, these results have been confirmed in another review study [ 28 ]. 

Among the strengths mentioned in the present study, the use of tetracycline ophthalmic ointment (1%) was used for lubrication, and the positive effect of this drug in reducing the microbial flora associated with implant treatment has been mentioned [ 29 - 30 ]. Most of the previous studies conducted on the topic were done *in vitro* and outside the patient's mouth. The clinical nature of the present study is one of its advantages over previous studies. One of the limitations of this study is the difficulty in following up the patients, which was resolved by the efforts of the study manager. It is recommended that more clinical studies be conducted on this subject to better determine and compare the effects of lubricants. 

## Conclusion

The study found no significant differences in detorque force values regarding gender or age. However, tetracycline eye ointment significantly increased the detorque force value compared to the non-lubricated group. These findings suggest its efficiency for enhancing implant-abutment stability in edentulous patients, though further studies are needed for a more reliable conclusion.
